# Fresh Pasta Manufactured with Fermented Whole Wheat Semolina: Physicochemical, Sensorial, and Nutritional Properties

**DOI:** 10.3390/foods8090422

**Published:** 2019-09-18

**Authors:** Simonetta Fois, Marco Campus, Piero Pasqualino Piu, Silvia Siliani, Manuela Sanna, Tonina Roggio, Pasquale Catzeddu

**Affiliations:** Porto Conte Ricerche Srl, Località Tramariglio, 07041 Alghero (SS), Italy

**Keywords:** sourdough, fiber, amino acids, phenolic compounds, phytic acid

## Abstract

Fresh pasta (SP) was prepared by mixing semolina with liquid sourdough, whole wheat semolina based, and the effects of sourdough inclusion were evaluated against a control sample (CP) prepared using semolina and whole wheat semolina. Physicochemical, nutritional, and sensorial analyses were performed on pasteurized fresh pasta, before and after cooking. The optimum cooking time was not affected by whole wheat sourdough, whereas differences were found in color, firmness, and cooking loss. Changes of in vitro digested starch fractions in SP pasta were affected by a higher cooking loss. Overall, SP samples were characterized by improved nutraceutical features, namely higher content of free essential amino acids and phenolic compounds, lower phytic acid content, and higher antioxidant activity. Sensory analyses (acceptability and check-all-that-apply (CATA) tests) showed significantly higher scores for the SP, and the differences were enhanced when the consumers were informed about the product composition and how it was manufactured. Consumers checked for more positive sensory parameters for the SP than the CP.

## 1. Introduction

Pasta is a staple food in the Mediterranean area, and it is produced and consumed worldwide. It is a good source of carbohydrates and proteins, with interesting nutritional properties, i.e., a low glycemic index. In recent years there has been a trend towards the production of whole wheat pasta, which represents a good source of fiber. The intake of dietary fiber exerts beneficial effects on human health. In fact, bran, a by-product of wheat milling, obtained from the outer layers of wheat kernel, contains fibers, vitamins, and minerals. Many advantages have been associated with the consumption of bran fiber, in terms of risk reduction of hypertension, breast cancer, and type 2 diabetes, and in terms of prevention of colon disease and gastric cancer [1,2]. Bran fiber is resistant to digestion and absorption at the small intestine level, and thus reaches the colon where bacterial fermentation occurs and produces short chain fatty acids [3]. On the other hand, wheat bran contains phytic acid, recognized as an anti-nutritional compound [4], which reduces the nutritional value by chelating ions (such as Ca^2+^, Fe^2+^, Mg^2+^, and Zn^2+^). Mineral deficiency could lead to decreased function of the immune system and reduced body growth and development [5]. With regards to bread making technology, the use of bran has some drawbacks which negatively affects volume, texture, and sensory acceptance [6]. Pasta prepared with the addition of bran has an inferior technological quality as compared with pasta prepared with semolina [7] or wheat flour [8], because bran interferes with gluten development, especially when bran presents inappropriately sized particles [9]. 

The general recognition of the positive nutritional effects of fiber consumption increase the demand for technological solutions to overcome the negative effects of bran supplementation. In bread making technology, fermentation of bran by microbial strains has been suggested as a method to reduce the negative effects of phytic acid [10] and to improve the volume and sensory properties of bread containing bran [11]. 

Few scientific papers have reported on the use of fermentation technology in pasta making. A gluten-free pasta was produced by [12] using buckwheat flour and 24 h fermented semolina, a vitamin B2-enriched pasta was produced by [13] using a 16 h prefermented semolina, and fresh pasta was produced by [14] using semolina and semolina-based liquid sourdough. In this study, fermented whole wheat semolina was used as a functional ingredient in pasta making, in order to ameliorate the detrimental effects of bran fraction over the structure and sensory features, while retaining the advantageous effects of bran on human health. Physical, chemical, sensorial, and nutritional characteristics were evaluated.

## 2. Materials and Methods

### 2.1. Raw Materials 

Commercial whole semolina (Integrale, Selezione Casillo S.r.l., Corato, Bari, Italy), and commercial semolina (Extra Arancio, Selezione Casillo S.r.l., Corato, Bari, Italy) were used in this study. The whole semolina had the following percent composition, as is or on the basis of dry matter (DM): moisture 14.1%, ash 1.6% DM, protein 12.5% DM, fiber 7.8% DM, dry gluten 8.5% DM, gluten index 60, alveographic W 199 (J × 10^−4^) and P to L ratio 5.12. The composition of semolina was the following: moisture 14.0%, ash 0.75% DM, protein 13% DM, fiber 2.7% DM, dry gluten 11% DM, gluten index 88, alveographic W 176 (J × 10^−4^) and P to L ratio 1.31.

### 2.2. Preparation and Maintenance of Liquid Whole Wheat Semolina-Based Sourdough (LWS)

A liquid whole wheat semolina-based sourdough (LWS) was prepared starting from the semolina-based liquid sourdough used by Fois et al. [14], which was refreshed for several days by back-slopping using whole wheat semolina and water, at a ratio of 1:1.5:1.5, in order to obtain a dough yield of 200. Back-slopping was done in the bioreactor GL MINI 25 (Esmach S.p.A., Grisignano di Zocco, Italy). The fermentation process was conducted at 26 °C for 5 h, and then LWS was kept at 5 °C until the subsequent daily back-slopping. The product was monitored daily in order to achieve stable values of pH and total titratable acidity (TTA). 

The ripe sourdough had a pH value of 4.3, a TTA of 12.4 mL NaOH (0.1 mol/L) in 10 g, and a viable cell number of approximately 10^7^ cfu/g for yeast and 10^5^ cfu/g for lactobacilli. Yeast cells were enumerated on Rose Bengal Chloramphenicol agar, and lactobacilli on de Man, Rogosa and Sharpe (MRS) modified agar medium [14]. Media and supplements were purchased from Oxoid (Basingstoke, England). 

### 2.3. Physicochemical Analyses of Pasta and LWS

A thermogravimetric analyzer Thermostep (Eltra GmbH, Haan, Germany) was used for moisture and ashes content determination, at 105 °C and at 600 °C respectively. Both TTA and pH were determined with an automatic titrator (Crison, Hach Lange, Barcelona, Spain), after homogenization of 10 g of sample in 90 mL of distilled water. After 30 min of gentle stirring for sourdough, and 60 min for pasta, the pH was determined, and the samples were titrated to a pH of 8.5 with NaOH 0.1 mol/L. The TTA was reported as mL of NaOH per 10 g of sample. The total dietary fiber (TDF) content of pasta was measured using the Total Dietary Fiber assay kit (Megazyme, Wicklow, Ireland). The color was determined on raw spaghetti placed side by side without any gap, using a CM-700d spectrophotometer (Konica Minolta, Osaka, Japan), using Standard Illuminant D65/10°. Prior to measurements, the instrument was calibrated against the white tile. CIE L*a*b* color space coordinates, lightness (L*), color in the red/green field (a*) and color in the blue/yellow field (b*), were computed. The Euclidean distance between colors, calculated as ΔE_76_, was used to estimate the range of perceived difference between samples of close chroma [15]:

(1) $$\Delta E_{76}=\sqrt{{\left(L_{2}^{*}-L_{1}^{*}\right)}^{2}+{\left(a_{2}^{*}-a_{1}^{*}\right)}^{2}+{\left(b_{2}^{*}+b_{1}^{*}\right)}^{2}}$$

0 < Δ76 < 1 the difference is unnoticeable; 1 < Δ76 < 2 the difference is only noticed by an experienced observer; 2 < Δ76 < 3.5 the difference is also noticed by an unexperienced observer; 3.5 < Δ76 < 5 the difference is clearly noticeable; 5 < Δ76 gives the impression that these are two different colors

Protein content (*N* × 5.27) was determined in 200 mg of pasta by the crude protein AACC (American Association of Cereal Chemists) combustion method 46–30 [16] using a Rapid N Cube analyzer (Elementar Analysensysteme GmbH, Langenselbold, Germany).

### 2.4. Pasta Making

The pasta was manufactured using the pasta maker “La Monferrina Dolly” (Moncalieri, Italy) equipped with a bronze die. The control pasta (CP) was prepared by mixing semolina (700 g) and whole wheat semolina (280 g) with 318 mL of water. Pasta with LWS, hereafter called sourdough pasta (SP), was prepared by mixing 38 mL of water, 700 g of semolina, and 560 g of LWS (consisting of 280 g of whole meal semolina and 280 mL of water). The dough was mixed for 20 min before extrusion into “spaghetti” of 3 mm diameter. After production, the CP and the SP were immediately pasteurized (at 97 °C for 3 min) and packaged under modified atmosphere (CO_2_:N_2_ = 30:70) [14]. For the analysis of total starch, protein digestibility, free amino acids, total phenolic compounds, antioxidant activity, and phytic acid, the samples were homogenized using a cryogenic mill (SpexSamplePrep, Stanmore, UK) and stored at −80 °C. All the analyses were replicated three times (*n* = 3).

### 2.5. Cooking Quality and Texture Analysis

The optimum cooking time (OCT), cooking loss (grams of solids in cooking water per 100 g of pasta as is), and swelling index (SI), i.e., grams of absorbed water per gram of pasta DM were measured for the CP and the SP according to the AACC Approved method 66–50 [16]. 

The texture of pasta, cooked to OCT, was evaluated according to the AACC Approved method 66–50 [16], using a TA.XTPlus Texture Analyzer (Stable Microsystems, Godalming, UK). The spaghetti strands were rinsed in cool water (4 °C) for 30 s to avoid overcooking. Tests were performed on 5 spaghetti strands, cut crosswise by the plexiglass blade probe A/LKB-F, at a test speed of 0.17 mm/s and a distance of 4.5 mm. The maximum force (*N*) of the curve, referred to as “firmness”, was computed. The software Texture Expert Exceed (v1.21) (Stable Microsystems, Godalming, UK) was used for texture data processing.

### 2.6. In Vitro Starch Digestibility

In vitro digestion of starch was performed on the CP and the SP, in order to quantify rapidly digestible starch (RDS), slowly digestible starch (SDS), and inaccessible digestible starch (IDS). RDS is the glucose released after 20 min of in vitro digestion. SDS and IDS are defined as the glucose released in the time frame between 20 and 120 min and between 120 and 180 min, respectively. IDS is defined as “inaccessible digestible starch” since it is not actually digestion-resistant starch, but just physically inaccessible to the digestive enzymes, and it was made accessible by homogenization of the sample after 120 min of in vitro digestion (Sanna et al., 2019).

Samples were cut 2 cm long and cooked to OCT, and then processed as in Sanna et al., [17].

### 2.7. Protein Digestibility and Free Amino Acid Analysis

The raw and cooked CP and SP samples were analyzed for protein digestibility [18,19] and free amino acid content. Free amino acid extract was prepared mixing 10 mL of 0.1 M hydrochloric acid solution and 1.5 g of homogenized sample, then, the mixture was vortexed for 10 s and centrifuged at 18,000× *g* at 4 °C for 15 min. Finally, the supernatant was filtered through a 0.22 μm PTFE syringe filter (Phenomenex, Macclesfield Cheshire, UK). The amino acid analysis was performed using an Agilent 1200 series HPLC system (Santa Clara, California, CA, USA), equipped with a binary pump with integrated vacuum degasser, an autosampler, a thermostated column compartment, and a diode array detector (DAD). The system was controlled by the Agilent Chemstation chromatography manager. The pre-column derivatization, gradient eluent method, and injection program were performed according to Agilent Application Note [20], using an Agilent Poroshell HPH-C18 column (4.6 × 100 mm, 2.7 µm pore size; Agilent Technologies, Santa Clara, California, USA) with HPH-C18 fast guard (Agilent Technologies, Santa Clara, California, USA), at a temperature of 40 °C. The pre-column derivatization was performed using *o*-phthalaldehyde reagent (OPA), 9-fluorenylmethyl chloroformate reagent (FMOC), and borate buffer supplied by Agilent Technologies. Standard solutions were purchased from Agilent Technologies, whereas GABA (γ-aminobutyric acid) was purchased from Sigma-Aldrich S.r.l. (Milano, Italy). Results were expressed as mg/100 g DM.

### 2.8. Total Phenolic Content and Antioxidant Activity

The total phenolic content (TPC) and 2,2-diphenyl-1-picrylhydrazyl (DPPH) radical scavenging activity were determined on raw and cooked CP and SP, by suspending 1 g of sample in 10 mL of an 80% aqueous methanol solution (20:80, *v*/*v*). The mixture was shaken for 2 h at 750 rpm in a thermomixer (Thermomixer Comfort, Eppendorf), then centrifuged at 800× *g* for 10 min. Supernatant was filtered through a 0.22 µm PTFE (Polytetrafluoroethylene) syringe filter (Phenomenex, Macclesfield Cheshire, UK) and stored at −20 °C until analyses. 

The TPC of sample extracts was determined using the Folin–Ciocalteau method [21] with the following modifications: 0.2 mL of the sample extract (or 80% methanol for the blank) was mixed with 1.5 mL of Folin–Ciocalteau reagent, previously diluted with water (1:10 *v*/*v*), and 1.5 mL of saturated sodium carbonate solution (7.5% *w*/*v*). The mixture was allowed to stand in the dark at room temperature for 1 h, then the absorbance was read at 735 nm, against the blank. The gallic acid calibration curve was built in the range 25–600 mg L^−1^ (y = 0.0051x + 0.071 *R*^2^ = 0.999) and results were expressed in terms of Gallic acid equivalents (GAE mg mL^−1^). 

The antioxidant activity of pasta was determined through the evaluation of free radical scavenging effect on the DPPH radical, according to [22] with some modifications: 1.4 mL of DPPH solution (0.10 mM in an 80% aqueous methanol) was mixed with 0.1 mL of sample extract, in a 1.5 mL centrifuge tube, vortexed, and then allowed to stand for 30 min in the dark. The discoloration of DPPH against the 80% aqueous methanol was monitored after 30 min, measuring the absorbance at 517 nm. The antioxidant activity of the sample was expressed as the percentage discoloration of DPPH solution, by the following equation:

% discoloration = [(AbsDPPH − AbsSample)/AbsDPPH] × 100(2)

where, Abs_DPPH_ is the absorbance of the DPPH solution without extract, and Abs_Sample_ is the absorbance of the sample solution after 30 min of reaction.

All reagents were analytical grade and purchased from Sigma-Aldrich S.r.l. (Milano, Italy).

### 2.9. Phytic Acid Determination

Phytic acid was determined on the raw and cooked CP and SP using the Phytic Acid (Phytate)/Total Phosphorus assay kit (Megazyme, Wicklow, Ireland). 

### 2.10. Consumer Testing

An acceptability test and a check-all-that-apply (CATA) method [23] were performed on the CP and SP samples. The test was carried out by 54 consumers, 26 women and 28 men, most of them recruited on the basis of interest and willingness. They were regular pasta consumers, aged between 32 and 60 years, not trained on sensory analysis of pasta products. Approximately, 40 g of packed sample were supplied to each consumer and the test was performed at their own home, under real conditions of use and consumption [24], so that consumers could season the pasta with the preferred sauce. The optimal cooking time, and the avoidance of spice and chili were suggested.

The evaluation of the SP and CP samples was carried out in two separate conditions, at two different sessions. The first condition was a blind test and the second condition was an informed test, in which consumers, before evaluating pasta, were informed on how the SP and CP pasta were produced. 

Consumers were asked to give a judgment for acceptability by scoring for the following attributes: flavor, taste, texture, and overall acceptability. A nine-point structured hedonic scale ranging from 1 (extremely disliked it) to 9 (extremely liked it) was used, and a sample was considered acceptable when it scored above 5 (neither like nor dislike). Then, consumers answered the CATA questionnaire, containing 12 phrases related to sensory, hedonic, and functional properties of the samples, which were the following: Do you believe that it is a wholesome food? Do you believe that it is ideal for a balanced diet? Do you feel it satiating? Do you sense a distinctive odor? Does it remind you of home-made pasta? Does it feel hard to chew? Do you feel it is tasty? Do you feel any unusual taste? Do you feel it “al dente” (cooked to OCT)? Does it absorb the sauce well? Do you feel it is sticky? Is it pleasantly sour?

The consumers were forced to answer “yes” or “no”, checking all phrases that applied as suitable to describe the product. 

### 2.11. Statistical Analysis

Standard ANOVA procedure (randomized complete design with three replicates and two treatments) was applied on the dataset. The means were separated by the LSD test at *p* = 0.05 significance level, using the Statgraphics Centurion software package (version16.1.11, Statpont Technologies Inc, Warrenton, VA, USA).

Hedonic scores collected in the acceptability test were analyzed by analysis of variance (ANOVA). The Cochran’s *Q* test at *p* = 0.05 was used to analyze the CATA data.

## 3. Results

### 3.1. Physicochemical Characteristics and Cooking Properties 

Physicochemical characteristics of samples are reported in Table 1. The fermentation process did not affect ashes, moisture, and protein content, which were similar in the SP and CP samples, nor the total dietary fiber content, which was measured prior to cooking. Moisture increased after cooking, similarly in the CP and SP, and this was obviously due to the water absorbed during cooking. Ashes decreased after cooking, probably because mineral salts were lost in the boiling water. After cooking, protein percentage increased in the CP, whereas it did not vary in the SP and probably this was an effect of cooking loss, as discussed later and displayed in Table 2. Cooking caused an increase in the pH of the SP and a decrease of the TTA values in the CP and SP, indicating that organic acids diffused into the cooking water. The lightness (L*) and the yellow color (b*) were significantly higher in the SP than in the CP, as showed in Table 1, suggesting a greater retention of the pigments which could contribute to a higher antioxidant activity [14,25]. The Euclidean color distance, ΔE_76_, was 4.26, depicting a clearly noticeable difference at human sight [15].

Table 2 shows the results of cooking quality analysis and texture of cooked pasta. The optimal cooking time was seven minutes for both the SP and the CP. The CP and SP showed the same swelling index, indicating the same amount of water absorbed, whereas differences were found in the cooking loss, which was significantly higher for the SP samples. Analysis of solids leached into the cooking water, indicate that the SP released more proteinaceous substances than the CP. Moreover, the SP showed a lower firmness than the CP. 

During the cooking of pasta, starch is normally leached into the water, together with soluble proteinaceous material. Kordonowy and Youngs [26] reported that the cooking loss was higher in bran-containing food, as a consequence of water-soluble components of bran and gluten dilution. In the SP the protein loss in the cooking water was higher than in the CP, probably because proteins have been partially hydrolyzed to peptides and amino acids by microbial proteases and, to a greater extent, by endogenous proteases and peptidases, which are active at a low pH. The proteolytic activity on the gluten proteins also explains the lower firmness of cooked pasta.

### 3.2. In Vitro Starch Digestibility 

**Figure 1:**
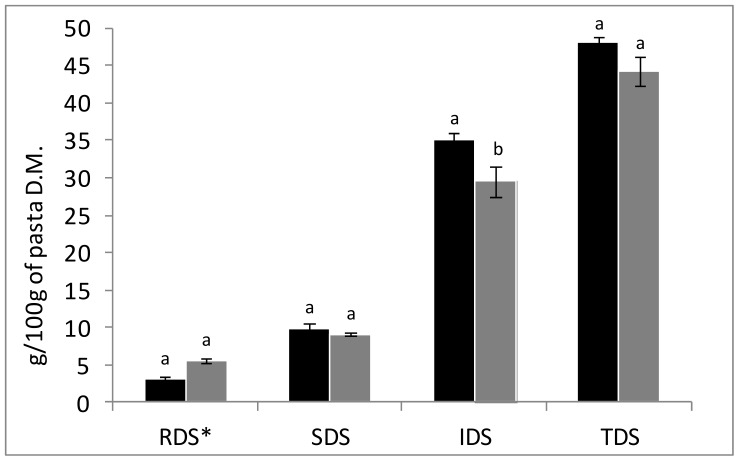
Results of in vitro digestion of pasta samples. RDS, rapidly digestible starch; SDS, slowly digestible starch; IDS, inaccessible digestible starch; TDS, total digestible starch. Black bars, CP and grey bars, SP. Different letters (a, b) for the same starch fraction denote a statistically significant difference between samples at *p* ≤ 0.05.

The results of glucose release after in vitro starch digestion and total starch values are reported in Figure 1. No significant differences were found between samples for the rapidly digestible starch (RDS), slowly digestible starch (SDS), and total digestible starch (TDS), whereas a significant difference was found for the inaccessible digestible starch (IDS), which resulted in being significantly lower in the SP sample. The differences observed for the TDS (44 g/100 g DM for the SP against 48 g/100 g DM for the CP), nevertheless not significant at *p* = 0.05, are fairly high. The acidic conditions of the SP before the heat treatment (pasteurization) should have been responsible for a stricter interaction between starch and gluten [27], and thus an increase of IDS, the starch fraction indigestible because of the food structure, was expected in the SP. Moreover, several studies have investigated the effects of dietary fiber in food and pasta on in vitro digestibility [28,29] showing that dietary fiber might have been responsible for the formation of a polysaccharide network that could encapsulate the starch granules during processing. The entrapment of starch reduces accessibility to enzymatic degradation, and therefore reduces the sugars released in the blood [30]. Sourdough technology applied to whole wheat bread already has been proved to retard postprandial glucose and insulin response of bread, with respect to yeast leavened whole wheat bread [31]. The decrease of IDS in the SP could be explained taking into account the higher cooking loss observed in the SP samples, which was a consequence of a greater disruption of food structure during cooking, thus altering the relative percentages of starch fractions. Sourdough determines the partial hydrolysis of proteins, causing a higher loss of peptides and amino acids in the cooking water and, at the same time, a weakening of the gluten matrix. A weaker gluten matrix is less able to entrap swollen starch granules during cooking, resulting in higher cooking loss of starch, which explains the lower content of some starch fractions in the SP.

### 3.3. Protein Digestibility and Amino Acid Content 

The digestibility of protein and the total protein availability (i.e., protein content*digestibility) are reported in Table 2**,** expressed as a percentage of the average of triplicate runs. The digestibility is significantly lower in the SP (83%) as compared with the CP (86%), and consequently the value of protein availability is lower in the SP. The method used to estimate protein digestibility [18] measures the pH drop, after 10 min of hydrolysis, of an aqueous protein suspension that has been adjusted to a pH of 8.0 using NaOH. During enzymatic hydrolysis, carboxyl (-COO^−^) and amino (-NH3^+^) groups are released from proteins. Protons, (H^+^), released into the surrounding reaction medium give rise to a decrease of the pH. The lower protein digestibility found in the SP is, therefore, due to the fact that part of the accessible peptidic bonds, which are the source of protons measured during the procedure, had already been broken by protease of the liquid sourdough. Moreover, as observed by Desai et al. [19] phenolic compounds (which are present at higher amount in the SP sample, as discussed later on) can contribute to a decrease in the protein digestibility. Therefore, pH drop method results must be carefully interpreted when they come out from the analysis of fermented products. 

Table 3 shows the quantity of the 20 individual free amino acids (FAAs) measured in raw and cooked CP and SP. Aspartic acid, glutamic acid, asparagine, arginine, alanine, GABA and tryptophan had the highest concentrations in all samples, whereas the least abundant FAAs were glutamine, histidine, methionine and isoleucine. In the SP, the mean value of total FAAs was 197.41 mg/100 g and 171.91 mg/100 g, raw and cooked, respectively. For the CP it was 159.65 and 153.39 mg/100 g, raw and cooked, respectively. The SP had the highest total FAA content, likely due to the proteolytic activity of the sourdough, and significant (*p* < 0.05) differences could be found between the SP and CP samples for all amino acids, except for glycine, alanine, tryptophan, lysine, and proline. Commonly, the amount of each FAA was higher in the SP than the CP, except for GABA, asparagine, and glutamine which were higher in CP. SP also had a significantly higher content of total free essential amino acids (EAAs).

These results show the important role of the microbial fermentation in order to obtain a higher content of FAAs and EAAs, since the acidic conditions of sourdough activated the cereal proteinases. Furthermore, microbial peptidases released small peptides and FAAs into the food matrix [32]. Such a phenomenon already has been observed in bread. Lappi et al., [31] found more solubilized and smaller molecular weight proteins and peptides in sourdough than in yeast bread. 

The high level of glutamic acid in the SP was an effect of microbial deamidation of glutamine [33]. This metabolic pathway plays an important role in pH homeostasis and acid resistance of microorganisms. As reported by Zhao et al. [34], a high level of glutamic acid can also result in a more pronounced food taste. Glutamic acid is a metabolic precursor of GABA, a non-protein amino acid naturally present in cereals in small quantities and having different health benefits. Numerous studies describe an increase of GABA in sourdough produced with selected lactic acid bacteria strains [35]. In this study a lower amount of GABA was found in the SP with respect to the CP, likely due to a consumption by the yeast [36]. Moreover, the decrease of asparagine observed in the SP as compared to the CP, also can be due to the yeast metabolism. These results confirm that microbial activity can affect nutritional properties and the taste of fresh pasta.

### 3.4. Total Phenolic Content, Antioxidant Activity, and Phytic Acid Content

The total phenolic content (TPC) and the antioxidant activity, measured as a percentage of discoloration of DPPH radical with respect to a blank sample, are reported in Table 4. In raw pasta, both the TPC and the antioxidant activity values were higher in the SP samples (37.0 mg/100 g pasta DM and 6.3%) than the CP samples (23.5 mg/100g pasta DM and 3.6%). It is known that fermentation improves the bioavailability of phenolic compounds and induces enhancement of antioxidant activity, through a mechanism reviewed by Hur et al. [37]. In addition, cooking had a positive effect on the TPC and antioxidant activity, which significantly increased in both the CP and SP. The increase of the TPC in pasta after cooking has already been reported in pasta enriched with bran fractions [38], and it was mainly ascribable to the increase of ferulic acid [39,40]. The increase of the antioxidant activity is consistent with the observed increase in the TPC, in agreement with Fares et al. [39], who stated that ferulic acid esters that are linked to cell walls are released by the pasta matrix during cooking and that they do not lose their antioxidant capacity, even after the hydrothermal treatment. Note that the combined effect of fermentation and cooking made the level of the TPC in the SP to be about three times higher than that of the CP sample, but the concomitant increase in antioxidant activity (from 6.33% to 7.83%) did not have such a high extent. This is probably because ferulic acid shows a relatively weak antiradical effect [41]. 

The amount of phytic acid was different between samples (Table 4), being lower in the SP as compared with the CP, both in raw and cooked samples. The phytic acid increased significantly after cooking, in both SP and in CP.

Bioavailability of essential nutrients, such as minerals and amino acids, is strongly reduced by the chelating properties of phytic acid in food stuff containing bran. Kordonowy and Youngs [26] found that the addition of bran in pasta increased the phytic acid content, and also observed the loss of phytic acid into the cooking water. The chelating properties of phytic acid can be inactivated at acidic conditions by the endogenous phytase of wheat [42]. In our work the significant reduction of phytic acid in the SP with respect to the CP was due to the acidic conditions generated by fermentation, and this is an important consequence from a nutritional point of view. 

### 3.5. Consumer Testing

**Figure 2:**
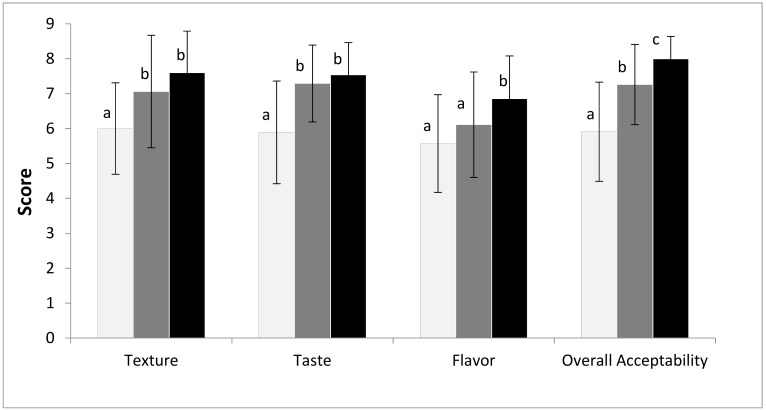
Results of the acceptability sensory test performed on the CP sample (white bars) and on the SP samples, the latter as blind (grey bars) and informed tests (black bars). Different letters (a, b, c) for the same sensory parameter denote a statistically significant difference between samples at *p* ≤ 0.05.

The main purpose of this study was to analyze how the use of fermented whole wheat semolina affected sensory properties of fresh pasta, taking into account that other authors reported lower sensory scores for high fiber pasta than for semolina pasta [7,26]. Figure 2 shows the results of the acceptability test performed on the CP and the SP, which was planned in two subsequent steps, the first one aiming to know the responses of consumers not informed on the SP properties (blind test), and the second one to know the responses of consumers informed on the preparation of the sample (informed test). As indicated in Figure 2, the scores of the CP sample were significantly lower for most of the sensory parameters as compared with those of the SP samples, except for the flavor which was similar to the value of the SP sample in the blind test but not in the informed test. Significant differences between the blind and informed tests, for SP samples, were found for flavor and overall acceptability, indicating the significant influence of information on food acceptability. Previous researches demonstrated that information on food stuff (brand, manufacturing, nutritional properties, etc.) might affect its hedonic rating [43,44].

Table 5 reports the relative frequencies of positive answers of consumers subjected to the CATA test. For the most part, the SP samples collect a greater number of positive responses than CP sample. Only the questions “Do you feel it hard to chew” and “Do you feel it sticky” collected a lower score and CP sample was found to be harder and more sticky than SP samples. To note that in the informed test the SP sample collected a greater number of positive responses than in the blind test, confirming that the information on the food preparation (i.e., the fermentation of semolina) had a positive impact on consumers’ expected food quality. The information provided on pasta influenced positively the “satiating” sensation, the “al dente” property and the ”absorption of sauce”, which obtained an higher score than the SP blind sample, whereas the “unusual taste”, observed in the blind sample, disappeared in the informed one. 

## 4. Conclusions

The addition of fermented whole wheat semolina affected many quality features of fresh pasta. Differences were found in color, firmness and cooking loss, while the optimal cooking time was the same for both samples. Notably, the SP samples were characterized by improved nutraceutical characteristics, showing a higher content in total and essential free amino acids, phenolic compounds, and resulting DPPH scavenging activity, and a decreased content of phytic acid. The results of sensorial analysis indicate an increase in the overall quality of pasta obtained using fermented whole wheat semolina, suggesting a new way for the sensorial improvement of high fiber pasta. The results of the acceptability test highlighted the differences between the CP and SP, with the latter having higher scores for all sensory parameters. The highest overall acceptability score was obtained from the SP sample after consumers were informed that the SP contained sourdough, indicating consumer interest in the addition of the functional ingredient. This study demonstrated that whole wheat sourdough is a valuable functional ingredient in fresh pasta making. Studies are in progress with in vivo trials, to investigate the nutraceutical properties of this innovative fresh pasta.

## Figures and Tables

**Table 1:** Physicochemical parameters of pasta. CP, control pasta; SP, pasta with sourdough. Mean values are reported.

	Ashes	Moisture	Protein Content	Dietary Fiber	pH	TTA	L	a	b	ΔE_76_
	g/100 g DM ^1^	g/100 g	g/100 g DM	g/100 g DM		mL NaOH 0.1*N*				
fermentation	ns	ns	ns	ns	***	***				
cooking	***	***	ns		***	***				
fermentation*cooking	ns	ns	**		***	***				
CP raw	0.97 ^a^	30.51 ^b^	13.75 ^b^	8.01	6.50 ^a^	2.48 ^b^	63.96 ^a^	2.45 ^a^	14.16 ^a^	
CP cooked	0.70 ^b^	58.24 ^a^	14.64 ^a^		6.65 ^a^	1.25 ^c^				4.26
SP raw	0.97 ^a^	31.01 ^b^	14.32 ^b^	8.16	5.11 ^c^	5.98 ^a^	68.01 ^b^	2.50 ^a^	15.46 ^b^	
SP cooked	0.61 ^b^	58.39 ^a^	13.72 ^b^		5.67 ^b^	2.45 ^b^				

Significance of the F-test after ANOVA: ns, not significant; **, significant for *p* ≤ 0.01; ***, significant for *p* ≤ 0.001. Different superscript letters for the same treatment denote a statistically significant difference at *p* ≤ 0.05. ^1^ DM, dry matter; TTA, total titratable acidity; L, lightness; a, color in the red/green field; b, color in the blue/yellow field; CP, control pasta; SP, sourdough pasta. ^a,b,c^ Means with different letters for each parameter indicate significant differences (*p* < 0.05).

**Table 2:** Cooking quality parameters, texture properties, and protein digestibility and availability of cooked pasta. CP, control pasta; SP, pasta with sourdough. Mean values are reported.

Cooked Samples	Cooking Loss * (%)	Swelling Index ** (g∙g^−1^)	Protein Loss in Water *** (%)	Firmness (*N*)	Protein Digestibility (%)	Protein Availability (%)
CP	4.61 ^a^	1.34 ^a^	0.36 ^a^	6.88 ^b^	86.6 ^b^	12.6 ^b^
SP	5.27 ^b^	1.36 ^a^	0.45 ^b^	5.64 ^a^	82.9 ^a^	11.9 ^a^

Different superscript letters for the same treatment denote a statistically significant difference at *p* ≤ 0.05. *, grams of solids in cooking water per 100 g of pasta (dry matter); **, grams of absorbed water per gram of pasta (dry matter); ***, g of proteins lost in water after cooking per 100 g of pasta (dry matter). ^a,b^ Means with different letters for each parameter indicate significant differences (*p* < 0.05).

**Table 3:** Free amino acid content in pasta samples (mg/100 g of pasta dry matter).

Aminoacids	Fermentation	Cooking	Fermentation*Cooking	CP		SP	
				Raw	Cooked	Raw	Cooked
Aspartic acid	**	***	ns	23.34	24.32	33.27	29.70
Glutamic acid	***	**	ns	14.86	15.19	29.48	26.36
Asparagine	***	***	*	21.63	20.62	14.86	13.41
Glutamine	***	***	***	4.84	5.24	1.24	1.34
Serine	***	***	*	3.03	2.77	5.62	5.41
Histidine	*	**	ns	2.22	2.13	2.84	2.46
Glycine	ns	***	ns	3.81	4.10	4.39	4.52
Threonine	***	***	ns	2.20	2.27	3.75	3.48
Arginine	***	**	ns	10.86	8.55	17.56	13.58
Alanine	ns	***	ns	11.15	11.43	12.11	11.52
GABA	***	***	ns	17.88	17.19	13.44	12.40
Tyrosine	*	**	ns	5.06	4.37	6.59	5.14
Valine	**	***	ns	7.78	7.39	10.86	9.82
Methionine	***	**	ns	0.68	0.70	1.31	1.23
Tryptophan	ns	**	ns	14.60	11.37	13.97	10.02
Phenylalanine	***	**	ns	3.36	2.96	6.26	4.81
Isoleucine	**	***	**	1.91	1.83	1.83	1.28
Leucine	***	**	ns	3.09	3.04	9.24	7.70
Lysine	ns	ns	ns	3.52	2.86	3.86	2.95
Proline	ns	***	ns	3.81	5.05	4.92	4.80
FAA ^1^	*	***	ns	159.65	153.39	197.41	171.91
EAA ^2^	**	**	ns	39.38	34.55	53.92	43.74

Significance of the *F*-test after ANOVA: ns, not significant; *, significant for *p* ≤ 0.05; **, significant for *p* ≤ 0.01; ***, significant for *p* ≤ 0.001. ^1^ FAA, free amino acids and ^2^ EAA, essential amino acids.

**Table 4:** Phytic acid and antioxidant activity in raw and cooked pasta. CP, control pasta and SP, pasta with sourdough.

Samples	Total Phenolic Content ^1^ (mg/100 g DM)	Antioxidant Activity ^2^ (%)	Phytic Acid (g/100 g DM)
fermentation	***	***	**
cooking	***	***	**
fermentation*cooking	***	**	ns
CP raw	23.53 ^c^	3.57 ^c^	0.26 ^a^
CP cooked	36.13 ^b^	6.53 ^b^	0.40 ^b^
SP raw	37.02 ^b^	6.33 ^b^	0.19 ^c^
SP cooked	104.87 ^a^	7.83 ^a^	0.25 ^d^

Significance of the F-test after ANOVA: ns, not significant; **, significant for *p* ≤ 0.01; ***, significant for *p* ≤ 0.001. Different superscript letters for the same treatment denote a statistically significant difference at *p* ≤ 0.05. ^1^ mg of gallic acid per 100 g of pasta (dry matter); ^2^ percentage of discoloration referred to blank sample. ^a,b,c^ Means with different letters for each parameter indicate significant differences (*p* < 0.05).

**Table 5:** Check-all-that-apply (CATA) test results. The reported values indicate the relative frequency of positive answers. In the first column the main attributes representing the questions addressed to the consumers, as follows: Do you believe that it is a wholesome food? Do you believe that it is ideal for a balanced diet? Do you feel it is satiating? Do you sense a distinctive odor? Does it remind you of home-made pasta? Does it feel hard to chew? Do you feel it is tasty? Do you feel any unusual taste? Do you feel it “al dente” (cooked to OCT)? Does it absorb the sauce well? Do you feel it is sticky? Is it pleasantly sour?

Attributes	Significance	CP	SP Blind	SP Informed
wholesome	***	0.29 ^a^	0.42 ^a^	0.90 ^b^
balanced diet	***	0.29 ^a^	0.42 ^a^	0.81 ^b^
satiating	***	0.46 ^a^	0.54 ^a^	0.77 ^b^
distinctive odor	***	0.29 ^a^	0.79 ^b^	0.98 ^b^
home made	***	0.37 ^a^	0.79 ^b^	0.98 ^b^
hard to chew	***	0.79 ^b^	0.21 ^a^	0.21 ^a^
tasteful	***	0.21 ^a^	0.81 ^b^	1.00 ^b^
unusual taste	***	0.04 ^a^	0.29 ^b^	0.034 ^a^
“al dente”	***	0.19 ^a^	0.50 ^b^	1.00 ^c^
adsorbs sauce	***	0.56 ^a^	0.77 ^b^	0.92 ^b^
sticky	***	0.33 ^b^	0.19 ^a,b^	0.00 ^a^
gently sour	***	0.00 ^a^	0.48 ^b^	0.71 ^c^

Significance of the Cochran *Q*-test ***, significant for *p* ≤ 0.001. Different superscript letters for the same treatment denote a statistically significant difference at *p* ≤ 0.05. ^a,b,c^ Means with different letters for each parameter indicate significant differences (*p* < 0.05).
